# Substitution spectra of SARS-CoV-2 genome from Pakistan reveals insights into the evolution of variants across the pandemic

**DOI:** 10.1038/s41598-023-48272-5

**Published:** 2023-11-28

**Authors:** Javaria Ashraf, Sayed Ali Raza Shah Bukhari, Akbar Kanji, Tulaib Iqbal, Maliha Yameen, Muhammad Imran Nisar, Waqasuddin Khan, Zahra Hasan

**Affiliations:** 1 Department of Pathology and Laboratory Medicine, Aga Khan University, Stadium Road, P.O. Box 3500, Karachi, 74800 Pakistan; 2 Department of Pediatrics and Child Health, Aga Khan University, Karachi, Pakistan; 3 Department of Pediatrics and Child Health, CITRIC Center for Bioinformatics and Computational Biology, Aga Khan University, Karachi, Pakistan

**Keywords:** Genetics, Molecular biology, Diseases, Health care

## Abstract

Changing morbidity and mortality due to COVID-19 across the pandemic has been linked with factors such as the emergence of SARS-CoV-2 variants and vaccination. Mutations in the Spike glycoprotein enhanced viral transmission and virulence. We investigated whether SARS-CoV-2 mutation rates and entropy were associated COVID-19 in Pakistan, before and after the introduction of vaccinations. We analyzed 1,705 SARS-CoV-2 genomes using the Augur phylogenetic pipeline. Substitution rates and entropy across the genome, and in the Spike glycoprotein were compared between 2020, 2021 and 2022 (as periods A, B and C). Mortality was greatest in B whilst cases were highest during C. In period A, G clades were predominant, and substitution rate was 5.25 × 10^–4^ per site per year. In B, Delta variants dominated, and substitution rates increased to 9.74 × 10^–4^. In C, Omicron variants led to substitution rates of 5.02 × 10^–4^. Genome-wide entropy was the highest during B particularly, at Spike E484K and K417N. During C, genome-wide mutations increased whilst entropy was reduced. Enhanced SARS-CoV-2 genome substitution rates were associated with a period when more virulent SARS-CoV-2 variants were prevalent. Reduced substitution rates and stabilization of genome entropy was subsequently evident when vaccinations were introduced. Whole genome entropy analysis can help predict virus evolution to guide public health interventions.

The acute respiratory disease, novel Coronavirus (COVID-19) outbreak in 2019 caused a global pandemic as the disease spread rapidly globally from Wuhan, China, affecting more than 209 countries including Pakistan^1,2^. It was caused by the coronavirus renamed Severe Acute Respiratory Syndrome Coronavirus 2 (SARS-CoV-2). COVID-19 has surpassed over 676 million cases, with 6.88 million deaths globally as of 10th March 2023 (https://coronavirus.jhu.edu/map).

High mutation rates can cause RNA viruses to become locally extinct^3^. Coronaviruses were previously thought to undergo fewer mutations compared to other RNA viruses; earlier outbreaks were caused by severe acute respiratory syndrome coronavirus (SARS–CoV) in 2002, Middle East respiratory syndrome coronavirus (MERS–CoV) in 2012 and the on-going pandemic of SARS–CoV-2^4,5^. The novel SARS-CoV-2 genome clustered with Sarbecovirus members as well as SARS-like coronaviruses obtained from bats, which caused the SARS pandemic in 2002–2003^6,7^. SARS-CoV-2 shared 79% similarity with SARS–CoV and 50% similarity with MERS–CoV^8^. The rate of mutations in SARS-CoV-2 was two times lower than MERS-CoV shown by Zhang et al. in 2016 and seven times lower than the mutation rate of SARS‐CoV^9,10^.

SARS-CoV-2 evolved quite rapidly through the pandemic, from the early Wuhan or S clade to G and GH clade strains^11^. Variants of Concern (VOC) emerged as more pathogenic and transmissible than wild type strains, causing pandemic surges. Virulence of VOC was associated with mutations in the Spike (S) surface glycoprotein which affected interaction of SARS-CoV-2 with host cells, impacted viral entry and transmission, and reduced neutralization by antibodies from previous infections^12^. The Alpha variant (B.1.1.7) was the first VOC found in late 2020 (https://www.cdc.gov/coronavirus/2019-ncov/variants/variant-info). This were followed by Beta (B.1.351), Gamma (P.1), Delta (B.1.617.1/2) and Omicron (B.1.1.529) variants, each characterized by unique signature mutations^13^. The Delta variant was 50 percent more transmissible than Alpha and found to escape host neutralizing antibody responses^14^. The Delta wave was surpassed by the Omicron (B.1.1.529) variant, which subsequently dominated all others globally by 2022^12,13^. The Omicron BA.2 lineage subvariant XBB, first identified in India in August 2022, became the most dominant by the end of 2022^15,16^. Phylogenetic analysis of Omicron variants in provinces across Pakistan revealed that BA.1 and BA.2 variants were first introduced in the province of Sindh^17^.

In Pakistan, 1.577 million COVID-19 cases were reported with 30,644 deaths (https://coronavirus.jhu.edu/region/pakistan) up to 6th March 2023. The first case was reported on 26^th^ of February 2020 and by 31 March 2020 there were 1,526 reported COVID-19 cases and 11 deaths, with a case fatality rate (CFR) of 0.7% (https://ourworldindata.org/coronavirus/country/pakistan). By 30 December 2021, 475,085 COVID-19 cases and 9,992 deaths had been reported with, bringing up the CFR to 2.1% (https://ourworldindata.org/coronavirus/country/pakistan). The Omicron wave was associated with a surge of cases from 1.29 million on 31 December 2021 to 1.57 million cases by 31 August 2022. The total number of COVID-19 associated deaths increased to 30,575 deaths but the CFR declined to 1.9% (https://ourworldindata.org/coronavirus/country/pakistan).

COVID-19 vaccinations were first introduced in Pakistan in February 2021 with a phase-wise approach, due to the limited availability of vaccinations. Health care workers and older age groups were vaccinated first, with subsequent age-wise availability in younger age groups. The vaccinations reduced COVID-19 severity and the disease burden^18,19^.

Investigations on the global evolution of SARS-CoV-2 by Li *et* al.^20^ during the first month of the pandemic estimated mutation rates to range between 1.7926 × 10^−3^ and 1.8266 × 10^−3^ substitution per site per year. In Africa, between February and March 2020, SARS-CoV-2 substitution rate was 4.133 × 10^−4^ per nucleotide per year^21^. Four months later, Shen *et* al. ^22^ used global data to show this was 3.95 × 10^−4^ per nucleotide per year^22^. In the USA, between February 2020 and March 2021, the substitution rate was 6.677 × 10^−4^ per site per year^23^. These data suggest variability between the SARS-CoV-2 mutation rates, viral evolution and pandemic surges. It is important to understand mutation rates in the regional context. Overall, COVID-19 morbidity and mortality rates were lower in Pakistan as compared with other regions. This could be attributable to several factors such as the relatively younger population, or prior immunity from cross-reactive antibodies^24^. Here, we investigated SARS-CoV-2 mutation rates and entropy across the genomes of Pakistan, during the period March 2020 until August 2022 to investigate the association between virus evolution and observed trends in COVID-19.

## Results

### COVID-19 waves and SARS-CoV-2 variants in Pakistan

Pakistan experienced two surges of COVID-19 each in 2020, 2021 and 2022, respectively. We investigated year-wise COVID-19 cases with mortality and SARS-CoV-2 variants across the study period March 2020 until August 2022. The trend of COVID-19 cases and deaths reported are shown in (Supplementary Fig. 1). We examined this period in three parts,** A** (10th March 2020–9th December 2020) started with the from 1st case reported in Pakistan until the introduction of Alpha variants^25^. The second period, **B** (10th December 2020–9th December 2021) covered the pandemic waves in 2021 associated with Alpha, Beta, Gamma and Delta variants in Pakistan^26,27^. The third period, **C** (10th December 2021–15th August 2022) associated with the Omicron wave in 2022^17^. Mortality due to COVID-19 increased at the beginning of the pandemic in 2020 (A) and then during 2021 (B), peaking in July. Subsequently, there was a decrease in mortality in 2022 (C) even though the COVID-19 cases surges at the beginning of the year.

**Figure 1:**
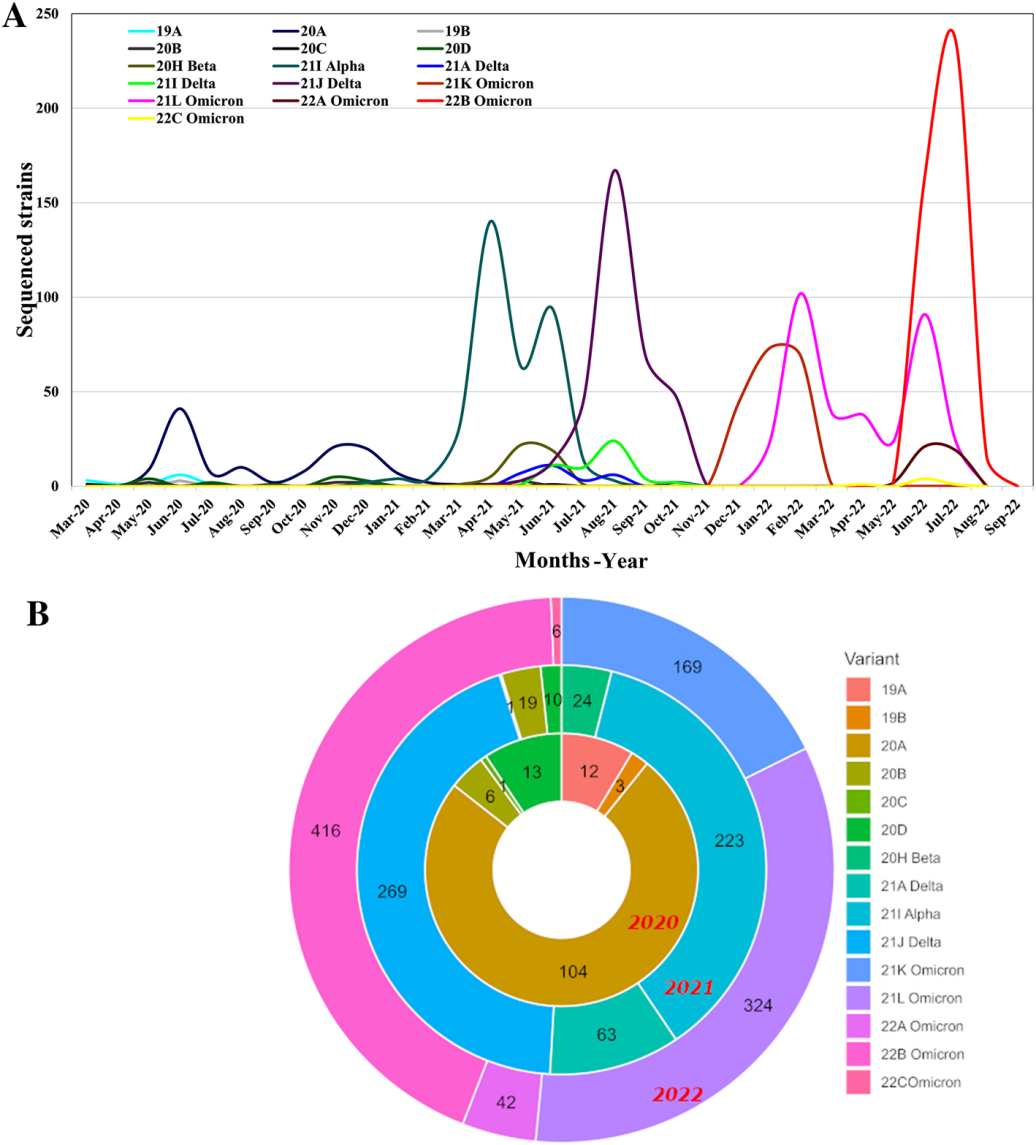
Emergence and dominance of different SARS-CoV-2 variants in Pakistan. More than 1,900 SARS-CoV-2 genome sequences from Pakistan were submitted in GISAID from March 2020 till August 2022. (**A**) The line graph depicts SARS-CoV-2 variants with the passage of time from 2020 till August 2022. (**B**) The chart represents the numbers of each variant used for phylogenetic analysis (n = 1705), between March 2020 and August 2022.

We determined the phylogeny of SARS-CoV-2 variants during the period. 1705 SARS-CoV-2 genomes were collected during this time of which clades ranged from 19A to 22C Omicron (Fig. 1A). The first strains introduced in March 2020 were 19A, followed by the emergence of 19B, 20A, 20B, 20C and 20D up until June 2020 after which these remained dominant till December 2020. The Alpha variant emerged in Pakistan at the end of December 2020, remaining dominant until July 2021. In 2021, Beta variants were observed in March 2021. The Delta variant emerged in June 2021 and remained dominant till December 2021. After December 2021, Omicron variants became predominant and are currently circulating to date (1 July 2023).

The overall number of SARS-CoV-2 samples sequenced throughout Pakistan for this period are depicted in the pie-chart (2020–2022), Fig. 1B. Genomes analyzed were A (n = 139), B (n = 609) and C (n = 957), Supplementary Table 1. The year-wise distribution of the genomes depicts the predominance of wildtype strains in period A, followed by Alpha and Delta variants in period B and Omicron variants in period C.

### Distribution of SARS-CoV-2 variants

Phylogenetic analysis was conducted using the Augur pipeline to further define the SARS-CoV-2 clades of each genome. Clade distribution in each  of the three study periods is depicted in Table 1. In A, dominant clades were 20A (55%), 20B (20%), 19A (13%), 20D (7%), 19B (4%) and 20C (1%). In B, most prevalent clades were 21 J Delta (44%) and 21I Alpha (37%) followed by, 21A Delta (10%), 20H Beta (4%) and 20D (2%). Period C comprised only of Omicron clades of which, the most prevalent were 22B Omicron (43%) and 21L Omicron (34%), and followed by 21 K Omicron (18%), 22A Omicron (4%) and 22C Omicron (1%).

**Table 1:** Description of SARS-CoV-2 genomes analyzed across the study period.

Clade/ Variants	SARS-CoV-2 genomes			% of total variants			% of the variant in the period
	A (N = 139)	B (N = 609)	C (N = 957)	A (%)	B (%)	C (%)	N = 1705(%)
19A	12	1	0	9	1	0	< 1
19B	3	0	0	2	0	0	< 1
20A	104	0	0	75	0	0	6
20B	6	19	0	4	2	0	2
20C	1	0	0	1	0	0	< 1
20D	13	10	0	9	2	0	1
20H Beta	0	24	0	0	4	0	1.5
21I Alpha	0	223	0	0	37	0	13
Delta	0	332	0	0	54	0	20
21A Delta	0	63	0	0	10	0	4
21 J Delta	0	269	0	0	44	0	16
Omicron	0	0	957	0	0	100	56
21 K Omicron	0	0	169	0	0	18	10
21L Omicron	0	0	324	0	0	34	19
22A Omicron	0	0	42	0	0	4	2.5
22B Omicron	0	0	416	0	0	43	24
22C Omicron	0	0	6	0	0	1	< 1

The table depicts the numbers of each SARS-CoV-2 genomes analyzed between March 2020 and August 2022 . Period A: 2020, B: 2021 and C: 2022. First column represents total variants reported. Second column represents the percentage of each variant within a specific period (A, B and C). The third column represents the percentage of each variant in three periods separately. Last column shows the percentage of each variant in periods (A + B + C).

### Substitution rates of SARS-CoV-2

**Figure 2:**
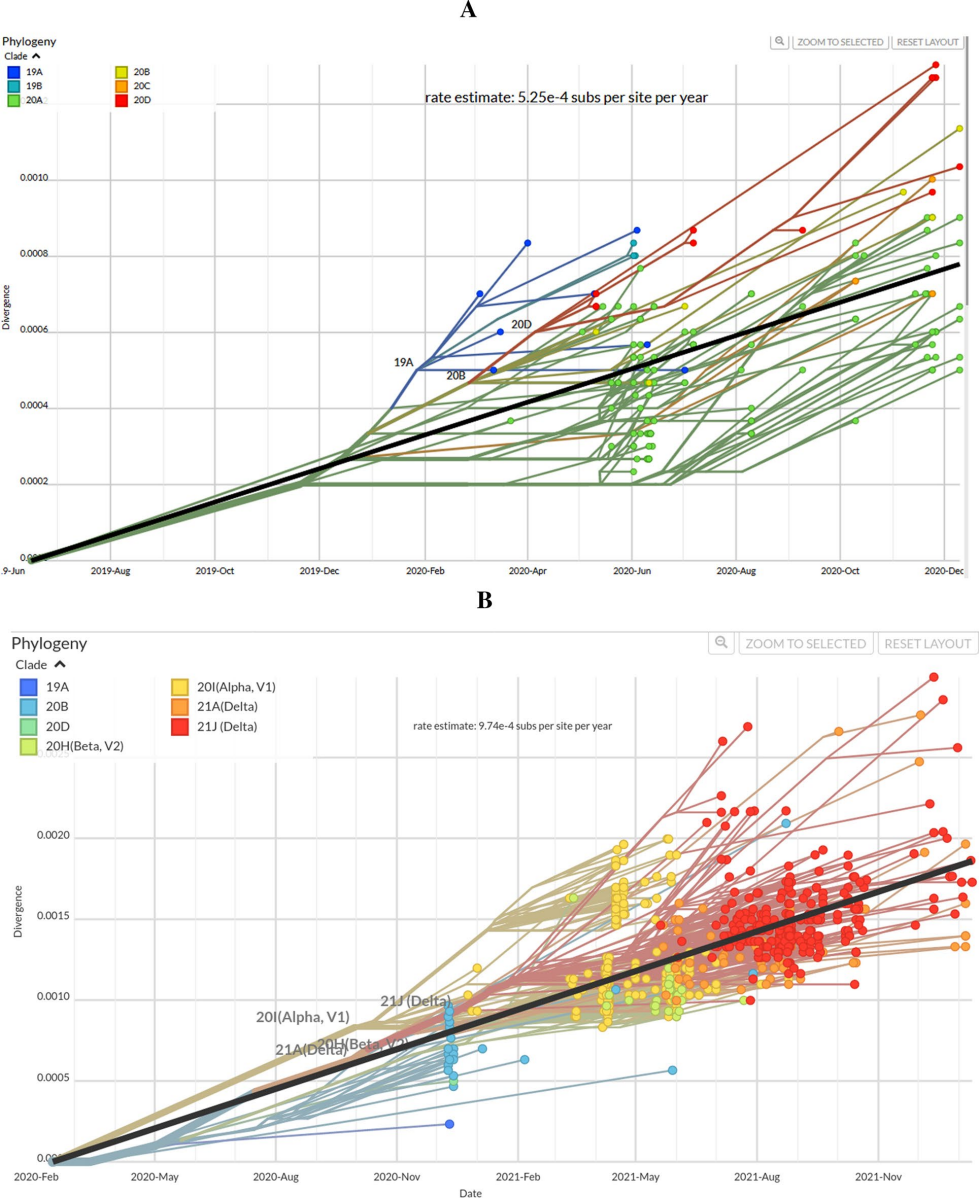

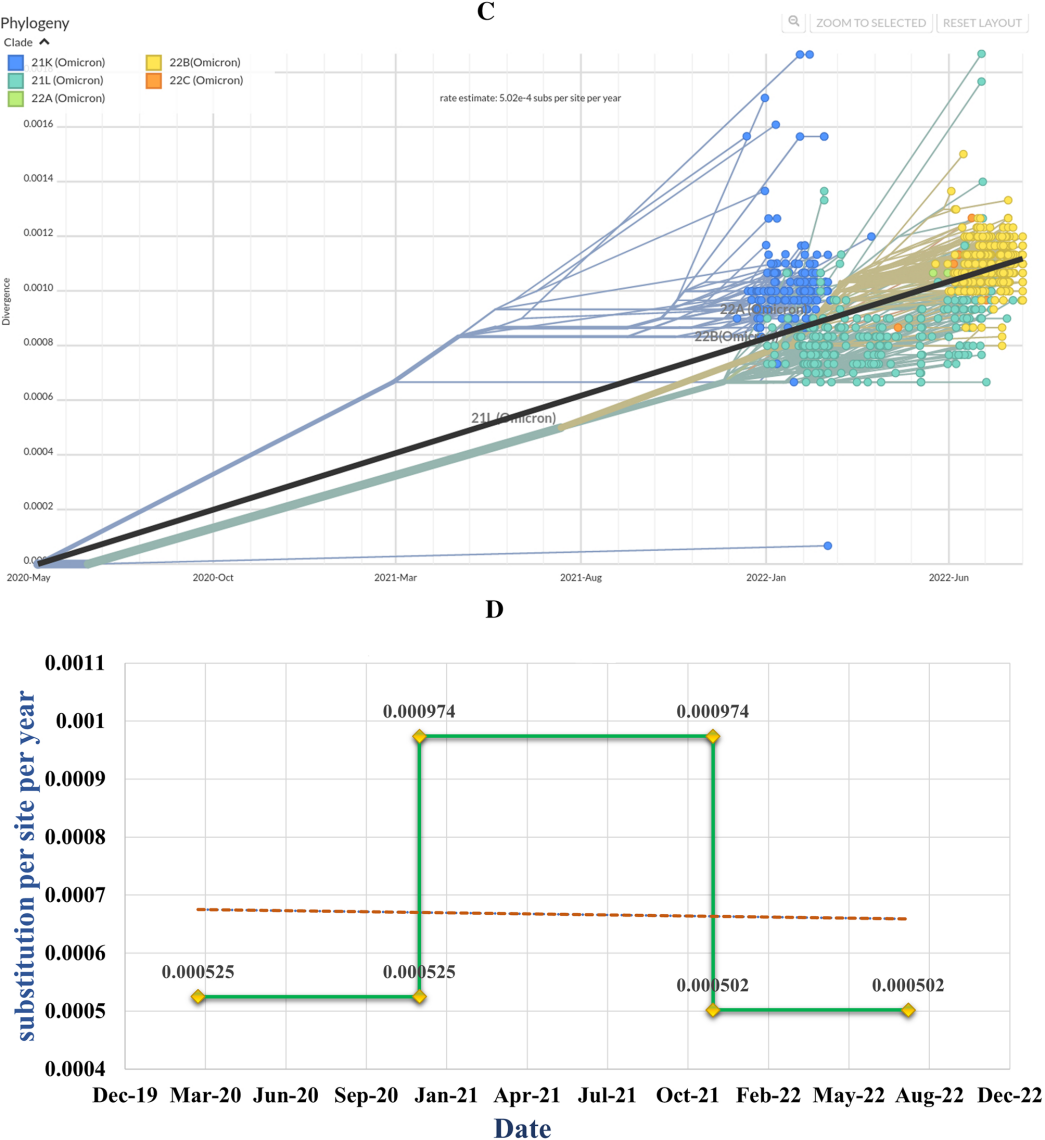
Substitution rate of SARS-CoV-2 genomes at different time intervals. (**A**) Phylogenetic analysis of SARS-CoV-2 sequences (period A) from 10th March 2020 to 9th December 2020. The tree was constructed using 139 sequences from all over Pakistan within the given time period. The clades are represented by distinct colors: 19A blue, 19B sky blue, 20A lemon green, 20B yellow, 20C orange and 20D red. (**B**) Phylogenetic analysis of SARS-CoV-2 sequences (period B) from 10th December 2020 to 9th December 2021. The tree was created using 609 sequences. New variants in this period were Alpha, Beta and Delta (21A, 21 J). (**C**) Phylogenetic Analysis of SARS-CoV2 sequences from 10th December 2021 to 15th August 2021. The tree was created using 957 sequences. The period showed new 21 K Omicron, 21L Omicron, 22A Omicron, 22B Omicron, and 22C Omicron. (**D**) Overall rate of mutation of SARS-CoV-2 (from March 2020 till August 2022) genomes from Pakistan.

We next investigated the genetic variation of SARS-CoV-2 isolates across the study period by determining their genome substitution rates. During A, the substitution rate was 5.25 × 10^–4^ subs per site per year (Fig. 2A). During B, the substitution rate was 9.27 × 10^–4^ subs per site per year (Fig. 2B). During C, the mutational rate decreased to 5.02 × 10^–4^ subs per site per year (Fig. 2C). Seen together, SARS-CoV-2 substitutions for three periods representing the waves of 2020, 2021 and 2022 revealed a high rate with the emergence of the novel pandemic virus, increasing during the period B (2021) (Fig. 2D), followed by a stabilization during C (2022) with a reduced rate of genome substitutions*.*

### Entropy analysis of SARS-CoV-2 sequences

Substitution patterns were calculated for each SARS-CoV-2 aligned genome with the reference sequence and observed over the period between 2020 and 2022. First average pairwise divergence was calculated, then compared with the proportion of C to U and G to U changes. Positional Entropy was calculated using Shannon entropy to quantify diversity at a single position^28^. We then investigated the relationship of positional entropy to any mutational hotspots in the SARS-CoV-2 genomes studied.

**Figure 3:**
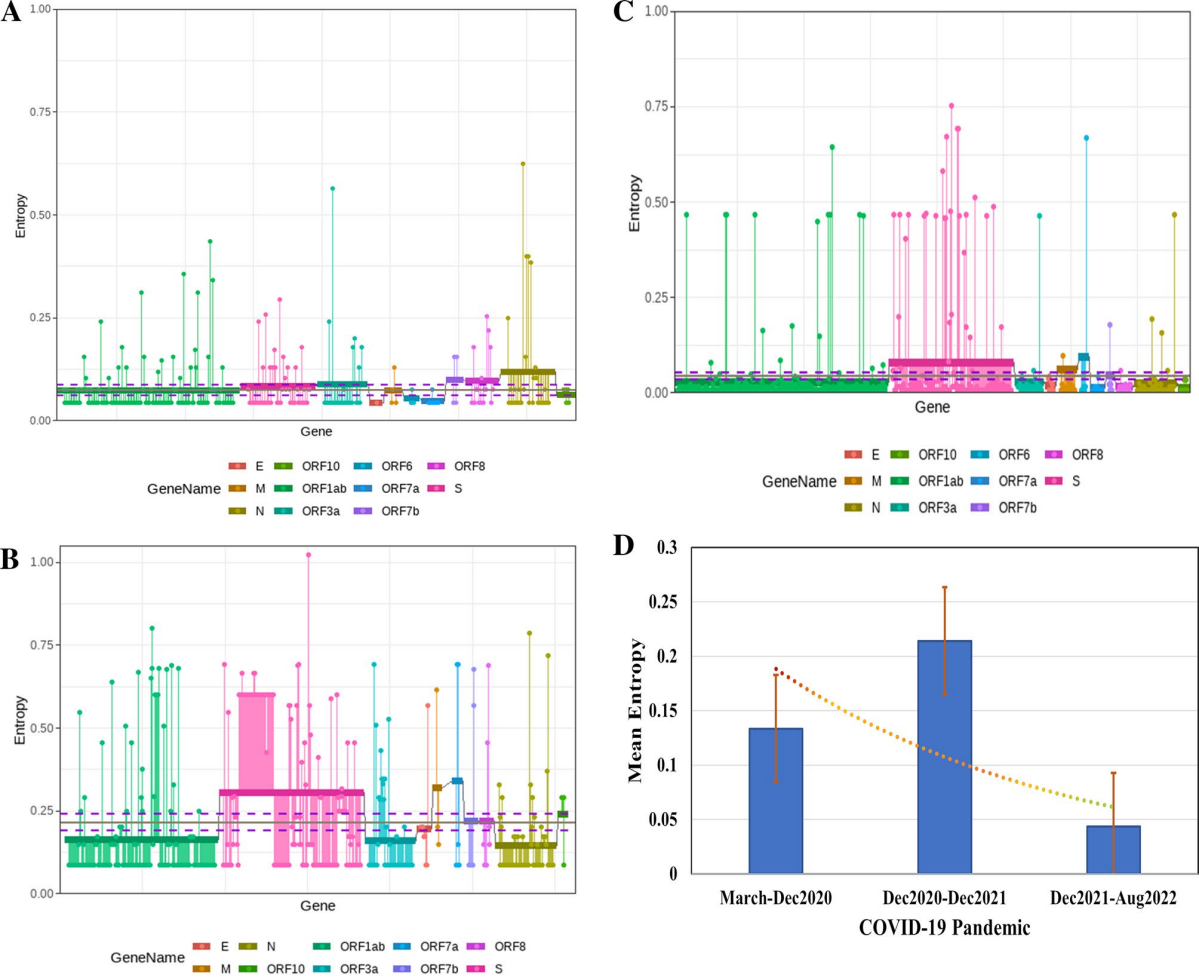
Exploring the positional entropy of SARS-CoV-2 genomes sequenced across the period. Positional Entropy is shown across 10 genes found in SARS-CoV-2 starting from ORF1ab to ORF10 highlighted by distinct colors (**A**) period A (10th March 2020 to 9th December 2020), (**B**) period B (10th December 2020 to 9th December 2021). (**C**) period C (10th December 2021 to 15th August 2022). (**D**) The mean entropy was calculated for all three periods; period A in blue, next bar is period C whilst the third bar shows period C (error bars in Orange).

We explored positional entropy through the analysis of 11 genes; ORF1ab, S, ORF3a, E, M, ORF6, ORF7a, ORF7b, ORF8, N and ORF10 (highlighted by distinct colors in Fig. 3). Positional entropy of the SARS-CoV-2 genomes during period A, when 19 (A, B) and 20 (A, B, C and D) clades were predominant, showed the most frequently mutated regions to be ORF1ab, N and Spike (S). No mutation was observed in the E gene region during period A (Fig. 3A).

During period B, when Alpha and then Delta strains were prevalent, higher rates of entropy were seen in S, followed by ORF1ab and M regions. The most frequent mutations were in the ORF1ab gene region, followed by S, N, ORF3a, ORF7b, ORF8, ORF7a, M, E and ORF10 (Fig. 3B)*.*

During period C, when Omicron variants were predominant, the rate of entropy was lower as compared with periods A and B. The most frequent mutations were in the ORF1ab gene region, followed by S, N, ORF3a, ORF6, M, ORF7a, ORF8, ORF7b, ORF8 and E (Fig. 3C).

### Mean entropy of SARS-CoV-2 sequences

The mean entropy of SARS-CoV-2 genomes across all three periods was calculated. The mean entropy was significantly varying in different time periods. Initially, the mean entropy was between 0.1 and 0.15 (Fig. 3D). During period B, there was a higher mean entropy, with values above 0.2. Overall, the highest mean entropy was seen in period B. In period C, the mean entropy was lowest.

### Associating regions of higher entropy with SARS-CoV-2 in different periods

We next determined if there was any association between the substitution rates of predominant variants and viral entropy during the study periods. We investigated positional entropy mainly of the S gene as its mutations have been associated with increased SARS-CoV-2 virulence, transmission, and vaccine escape^29^. Key S gene mutations include, K417N, E484K, N501Y, A701V, L452R, T478K and P681R^29–33^. There was a changing trend in the entropy site specific mutations during the study period. The S gene mutations K417N, L452R, T478K and A701V were absent in period A, E484K, N501Y, and P681R had entropy values of 0.043, 0.173 and 0.104 respectively (Table 2). However, all mutations were present in SARS-CoV-2 strains of period B; K417N, L452R, T478K, E484K, N501Y, P681R and A701V demonstrated entropy values of 0.185, 0.691, 0.69, 0.325, 0.7, 0.922 and 0.175, respectively. Hence, the highest entropy during period B was observed for P681R followed by that of N501Y, L452R and T478K. In period C, all mentioned mutations displayed lower entropy values except for L452R with entropy value of 0.753; K417N, T478K, E484K, N501Y, P681R and A701V showed value of 0.06, 0.068, 0.021, 0.021, 0.008 and 0.058, respectively. The highest entropy during period C was recorded for L452R followed by T484K, K417N, A701V and T484K, whilst N501Y and P681R showed the lowest entropy values.

**Table 2:** Varying entropy of mutations in Spike protein between 2020 and 2022.

Spike mutation	Entropy during A	Entropy during B	Entropy during C
K417N	Absent	0.567	0.048
L452R	Absent	0.691	0.753
T478K	Absent	0.692	0.048
E484K	0.043	0.233	0.021
N501Y	0.173	0.397	0.015
P681R	0.104	1.022	0.015
A701V	Absent	0.567	0.058

The table depicts the entropy of mutations in the Spike protein during the periods A (2020), B (2021) and C (2022).

The higher positional entropy of S gene mutations was found in Delta and Beta variants (period B) whereas, the lower entropy was seen in Omicron variants (period C).

## Discussion

Our study expands on the observations of SARS-CoV-2 variants and their diversity in Pakistan across the pandemic and links these with virus evolution through the study of genome substitution rates. This is the first extensive study on substitutions rates and entropy of genomes from Pakistan. We show that SARS-CoV-2 genome substitution rates rose during 2020, increasing further during 2021 followed by a decrease in 2022. The mean entropy across genomes increased in 2020 followed by decreases in 2021 and 2022. Study of positional entropy revealed key S gene mutations such as, K417T, L452R, T478K, E484K, N501Y, P681R and A701V, in variants. Our data can be associated with VOC related COVID-19 case and mortality rates.

We observed the change between clade 19A in 2020, to Alpha and Delta variants in 2021 and to Omicron variants in 2022. Our analysis agrees with previous reports showing; G and GH clade strains were predominant in 2020^11^ and Alpha variants were introduced in December 2020^34^. That the Beta variant dominated in March 2021 followed by a Delta variant peak in June 2021, after which Omicron variants surged in January 2022^26^. Although the Omicron variant peak in January 2022 in Pakistan led to the highest number of cases since the beginning of the pandemic^35,36^, a higher CFR was reported during the Delta wave of 2021 as compared with the Omicron wave of 2022 (https://covid.gov.pk/).

In 2020 it was reported that the original Wuhan strain of SARS-CoV-2 had acquired 27 proteins mutations within an year^37^. Mutations reported in the S region of the virus and were linked to transmissibility and disease severity^33,38^. Identification of S gene mutations led to the discovery of new SARS‐CoV-2 variants^39^. Some mutations related to enhanced binding efficiency to the angiotensin-converting enzyme 2 (ACE2) receptor, providing a selective advantage to the VOCs; leading to increased viral loads, transmission and virulence^30^. By considering mutations across the genome and not just in S protein, our study gives an additional perspective regarding diversity of SARS-CoV-2.

In 2021, the phylodynamic analysis platform Nextstrain predicted the annual nucleotide evolution rate of SARS-CoV-2 to be 8 × 10^−4^ substitution per nucleotide per year^40^. This is the first extensive study on substitutions rates and entropy of genomes from Pakistan. The substitution rate we observed for 2020 was higher than the 3.95 × 10^−4^ per nucleotide per year observed in the USA, after few months of outbreak^5^ and also in Africa, during the early pandemic period^21^. In the USA, the substitution rate was found between February 2020 and March 2021, to be 6.677 × 10^−4^ per site per year^23^. Ghanchi et al., calculated a substitution rate 5.68 × 10^−4^ per nucleotide per year in 2020, using 71 sequences from Pakistan collected between March and October 2020^11^. We observed a higher substitution rate (5.25 × 10^−4^ per nucleotide per year) for 2020 likely due to inclusion of almost twice as many genomes across a longer period (March until December 2020).  Reflects increasing entropy associated with G and GH clade strains as reported previously^11^. For 2021, we found SARS-CoV-2 genomes to display a 9.27 × 10^−4^ substitutions per site per year, fitting with the prediction made by Nextstrain^40^.

In 2022, the period during which the Delta variant was overtaken by Omicron wfas accompanied by a decrease in substitution rates during the latter period, suggesting that the genome was moving towards stability. Omicron variants have twice the number of mutations as compared with Delta^41^. Increased mutation at sites in the Omicron variant included, K417N, L452R, E484K, and N501Y which were related to ACE2 binding residues of SARS-CoV-2^41,42^.

Entropy helps to find disorder or abnormality in a system which can be used for signature identification to understand the conservation of amino acids at a specific location. Genome entropy was the highest during 2020, but with few mutation sites across the SARS-CoV-2 genome. In period 2021, the entropy remained high but involving more variable mutations sites, Further decline in entropy was observed in 2022, coincident with the Omicron surge, lower COVID-19 mortality rates, less viral pathogenicity but with higher infectivity rate (https://www.healthdata.org/sites/default/files/covid_briefs/165_briefing_Pakistan.pdf). A higher value of entropy in the viral genome is indicative of increased randomness at that site whereas lower entropy at the same sites is indicative of an increased stability and decreased randomness^43,44^. The specific S variants (K417N, E484K, N501Y, A701V) are reported to successfully evade the humoral immune response^30,44^. In our genome analysis, the S mutation with highest entropy was P681R in 2021, this substitution was associated with improved Spike protein cleavage of Delta variant, eventually increasing viral replication and cell fusion. Entropy at all the sites was greater in 2021 than in 2022 except for L452R site. The S gene L452R mutation entropy rates were high in both 2021 and 2022. L452R, is an RBD domain mutation associated with higher infectivity or transmissibility of SARS-CoV-2 in these periods, especially in period 2022^31^. During 2021, virus was more pathogenic likely associated with hotspot mutation regions, whereas Omicron variants were more transmissible in population due to higher entropy of key mutation L452R. Our data correlates with reports by Santoni *et* al. SARS-CoV-2 genome entropy of 17,271 strains from India during between the period March 2020 to July 2021; who observed an increase in average entropy particularly in the structural proteins during the Delta wave, which subsequently declined afterwards, when the genomes reached some stability^45^.

We observed the period in 2021 to be associated with increased substitution rates. There were Alpha and Delta variant surges^46,47^, with a cluster of variants (19A, 20B, 20D, 20H Beta, 20I Alpha, 21A Delta and 21 J Delta), causing the highest mortality, pathogenicity and infectivity rate globally (https://publichealth.jhu.edu/2022/covid-year-in-review).

Delta was the dominant VOC that driveg increases in infections, even in populations with high levels of access to vaccines^13^. The Omicron variant had lower entropy, which may have resulted in reduced severity and mortality, but mutations in the S region affected its transmissibility, increasing it by 2–7 fold as compared to Alpha, Beta and Delta^41,42^. The lower CFR from infections with Omicron as compared with Delta, despite the higher rate of new infections fits with other studies^48,49^. A study from our center has shown that patients infected with Omicron variant had lesser risk of severe disease and in-hospital mortality from COVID-19 as compared with non-Omicron variants, matching previous reports^50^.

Vaccines play their role in controlling the spread and their development and dissemination have brought rays of hope against the circulation of the different variants of SARS-CoV-2^]^. COVID-19 vaccination led to reduced transmission of the highly virulent Delta variant, followed by the introduction of Omicron variants which escape antibody mediated responses from the host but are cause less severe disease^53^. Globally, vaccination effectiveness against COVID-19 was found to be 92% against hospitalization and 91% for mortality^51^. Vaccines considerably reduced COVID-19 disease severity during pandemic surges caused by Delta and Omicron variants^18^.

Vaccinations were introduced in Pakistan during February 2021 in a stratified manner starting with healthcare workers and older age groups. It was reported that by July 2021, only 18.9% of Pakistani population had received COVID-19 vaccinations^54^. By December 2021 this rose to 31.3% (156.62 million vaccination doses were administered), and then to 55.9% by December 2022 (317.70 million vaccination doses were administered), https://ourworldindata.org/coronavirus/country/pakistan.

A limitation of our study was the relatively limited number of genomes available from Pakistan. Further, there was a difference in the number of SARS-CoV-2 genomes available for analysis representing the different periods A, B and C (2020, 2021 and 2022). The lesser number of genomes in the earlier periods especially during A, was related to scarcity of genomic sequencing capacity in laboratories including, difficulty in accessing reagents, limited scientific infrastructure and training for personnel. Further, period C was until August 2022 and did not cover the full year. However, as COVID-19 numbers were reduced by August 2022, we believe we had sufficient data for a comparison across 2020–2022. Overall, the relatively small number of SARS-CoV-2 genomes sequenced available for a country of 220 million people reflects the limited availability of infrastructure for genome sequencing studies. Unfortunately, we did not have vaccination data for the individuals from whom SARS-CoV-2 genomes were isolated. Therefore, we could not make a direct association between substitution rates and genome entropy due to vaccinations. However, information regarding vaccination rates and the VOCs causing different pandemic is available. Hence, the population was mostly unvaccinated in periods A and B, when SARS-CoV-2 genome substitution rates and entropy was higher entropy. Followed by period C, when substitution rates were lower and genomic entropy showed stabilization.

Overall, our study is an important one as it provides insights into the evolving genome of SARS-CoV-2 and demonstrates how substitution rate per site per year can be related to interpretation of genome entropy, positional and site-specific. As increased mutations in key genes are associated with viral surges, understanding the genome variance can help us understand when variants may result in higher infectivity in population or may result in more stability and adaptation to the host genome. Further work is required to understand whether this is directly related to vaccinations or other factors related to changes in viral genome diversity.

## Methods

This study was approved by the Ethical Review Committee, The Aga Khan University (AKU), Pakistan.

### Data used in this study

The COVID-19 case numbers presented in this study were obtained from the John Hopkins Coronavirus resource center which collects data from the official website of each country as mentioned in the article by Dong et al.^55^. The source data is the Pakistan Government official COVID-19 website, http://covid.pastic.gov.pk/national.aspxstats/pakistan.

### Data collection

A total of 2,089 full-length SARS-CoV-2 genomes and corresponding metadata of sequences submitted from Pakistan were downloaded from GISAID (www.gisaid.org), Supplementary Table 1. The collection dates of these samples ranged between March 2020 and August 2022. Sequences with low coverage were excluded. Sequence data was stratified into three periods A (10th March–9th December 2020), B (10th December 2020–9th December 2021) and C (10th December 2021–15th August 2022). For each period, the number of sequences were A, 140; B, 981 and C, 1022 respectively. Sequences with missing information were excluded. The final number of genomes analyzed across the period of the study were A,139, B, 609 and C, 957 which accumulated to a total of n = 1,705.

### Data processing

**Figure 4:**
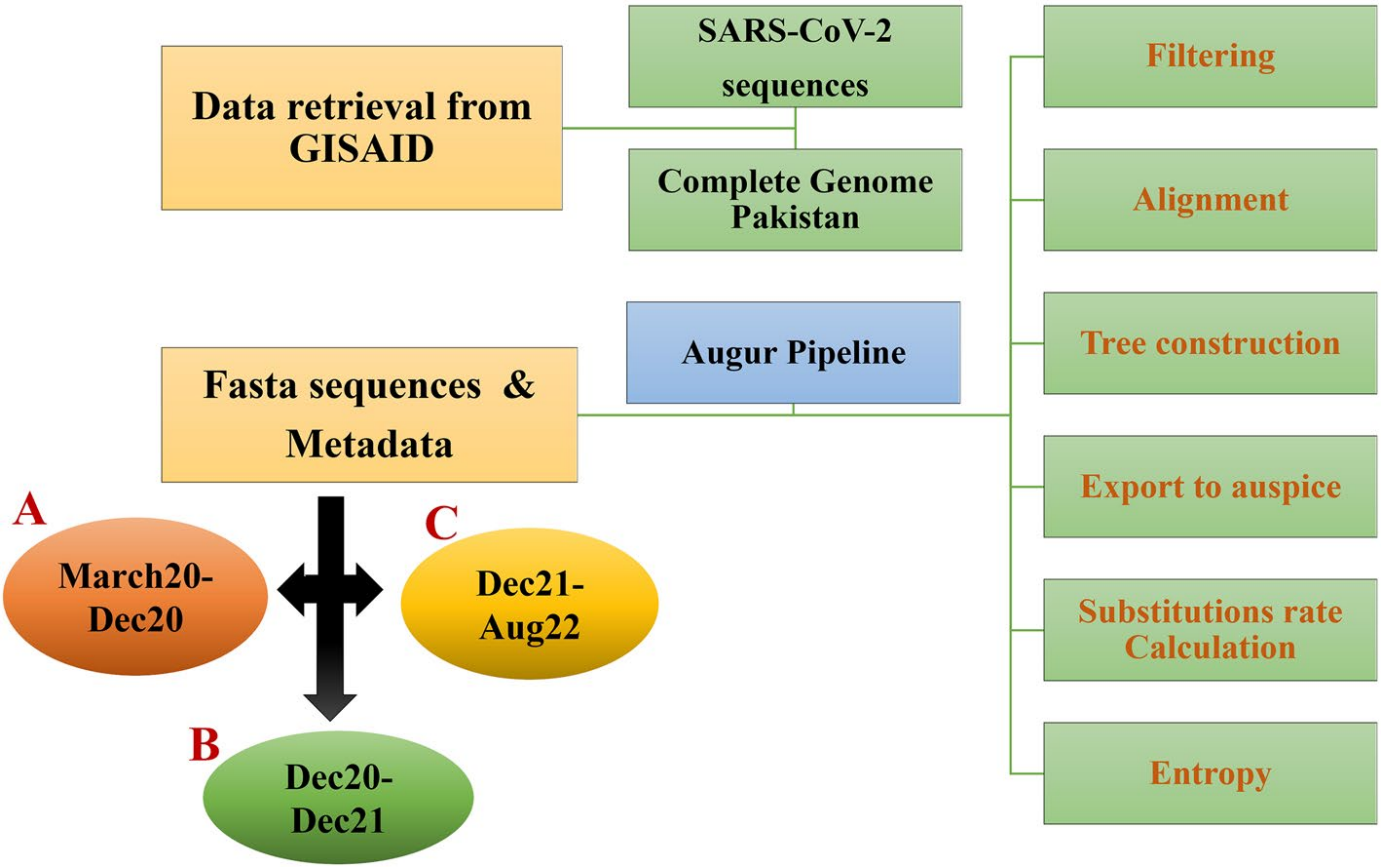
Flowchart depicting main steps of methodology followed in the phylogenetic analysis and substitutions rate calculation. A total of 1,705 complete SARS-CoV-2 genome sequences from Pakistan were downloaded from March 2020 till August 2022. Phylogenetic analysis using augur pipeline was applied for maximum likelihood tree construction, substitution rate and entropy calculation.

The Augur pipeline was followed to prepare datasets for phylogenetic analysis, substitution rates and entropy calculation^40^. Data analysis was performed as described in Fig. 4. Sequences were first filtered on the basis of country (Pakistan) and date (year and month), then aligned with respect to the reference sequence (NC 045,512.2)^56^ using the MAFFT^57^ alignment tool. An alignment maximum-likelihood tree was generated using iqtree2 tool by applying the substitution model General Time Reversible (GTR)^58^. The tree was further refine based on ancestral sequence inference and dating^59^. Augur pipeline was further used to assign clades to the nodes on the tree and infer ancestral sequences, phylogenetic tree was used to calculate branch-lengths, nucleotides and amino-acids substitution rate.

Shannon entropy such as defined as in an entropy-based study by Santoni et al.^45^. Entropy can be defined as:

$$E= -\left(\sum {d}_{x}{\mathrm{log}}_{2}{d}_{x}\right)$$

    Here in the above equation E is entropy, where ***d***_***x***_ is the probability of the *x*th value of a random variable. On the basis of arrangement of amino acids within each column of SARS-CoV-2 alignment with input sequences, we can measure the probability of occurrence of amino acids (*x* = 1–20) detected from each position of the aligned sequences with the SARS-CoV-2. Raised entropy was determined based on 2 standard errors of mean entropy of all sequences across the genomes within each period.

### Ethical approval

This study received approval from the Ethical Review Committee of the Aga Khan University (2022-6871-22203).

### Supplementary Information

Supplementary Figure 1.

Supplementary Table 1.

Supplementary Table 2.

## Data Availability

The data is downloaded from GISAID, and the supplementary Tables 1 and 2 list the data used in the article.
