# Supplementary material for: Substitution spectra of SARS-CoV-2 genome from Pakistan reveals insights into the evolution of variants across the pandemic
Source: Sci Rep. 2023 Nov 28;13:20955. doi: 10.1038/s41598-023-48272-5 (PMC10684861; doi:10.1038/s41598-023-48272-5)
Supplement: Supplementary file 1 — Supplementary Figure 1. [file 41598_2023_48272_MOESM1_ESM.pdf]

Title: Substitution spectra of SARS-CoV-2 genome from Pakistan reveals insights into the evolution of variants across the pandemic

- Javaria Ashraf[1],
- Sayed Ali Raza Shah Bukhari[1],
- Akbar Kanji[1],
- Tulaib Iqbal[1],
- Maliha Yameen[1],
- Muhammad Imran Nisar[2,3],
- Waqasuddin Khan[2,3] and
- Zahra Hasan[1]

[1]Department of Pathology and Laboratory Medicine, Aga Khan University, Karachi, Pakistan

[2]Department of Pediatrics and Child Health, Aga Khan University, Karachi, Pakistan

[3]CITRIC Center for Bioinformatics and Computational Biology, Department of Pediatrics and Child Health, Aga Khan University, Karachi, Pakistan

***Corresponding author**

Zahra Hasan, PhD, Professor, Department of Pathology and Laboratory Medicine, Aga Khan University, Stadium Road, P.O. Box 3500, Karachi 74800, Pakistan.

Email: zahra.hasan@aku.edu

Supplementary Figure 1. Description of COVID-19 cases and deaths in Pakistan. The graphs depict data for COVID-19 between 1st March 2020 and 15th August 2022. **Upper panel**, Cases reported and **Lower panel**, Deaths reported. Source, Our World in Data https://ourworldindata.org/. Data is stratified into three periods separated by dotted lines, **A** (10th March to 9th December 2020), **B** (10th December 2020 to 9th December 2021) and **C** (10th December, 2021 to 15th August, 2022). Arrow indicates the administration of vaccination.

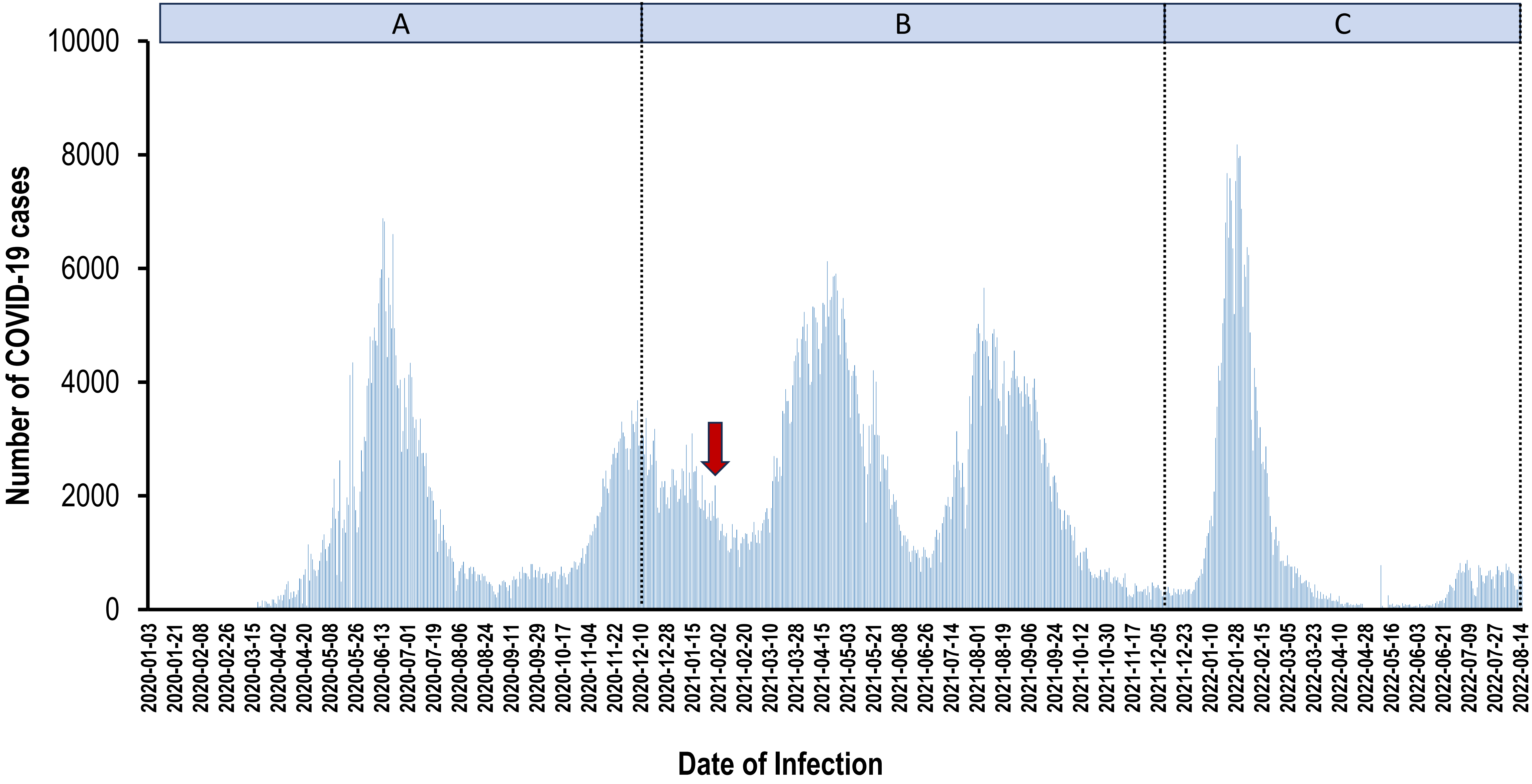

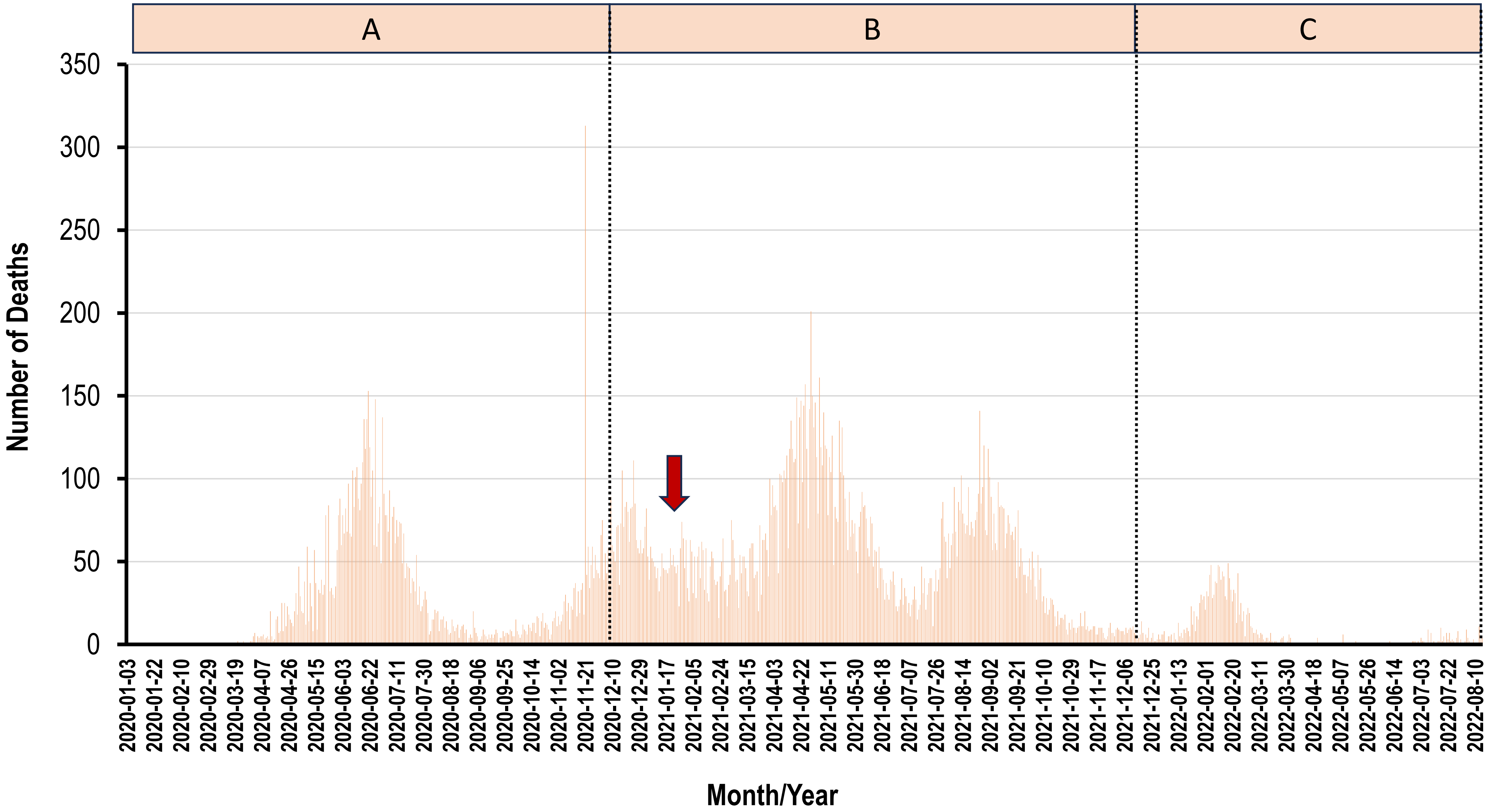

A
B
C
Number of Deaths
350
300
250
200
150
100
50
0
2020-01-03
2020-01-22
2020-02-10
2020-02-29
2020-03-19
2020-04-07
2020-04-26
2020-05-15
2020-06-03
2020-06-22
2020-07-11
2020-07-30
2020-08-18
2020-09-06
2020-09-25
2020-10-14
2020-11-02
2020-11-21
2020-12-10
2020-12-29
2021-01-17
2021-02-05
2021-02-24
2021-03-15
2021-04-03
2021-04-22
2021-05-11
2021-05-30
2021-06-18
2021-07-07
2021-07-26
2021-08-14
2021-09-02
2021-09-21
2021-10-10
2021-10-29
2021-11-17
2021-12-06
2021-12-25
2022-01-13
2022-02-01
2022-02-20
2022-03-11
2022-03-30
2022-04-18
2022-05-07
2022-05-26
2022-06-14
2022-07-03
2022-07-22
2022-08-10
Month/Year
